# Feasibility and preliminary experience of single-incision plus one-port laparoscopic total gastrectomy with Overlap esophagojejunostomy for gastric cancer: A study of 10 cases

**DOI:** 10.3389/fsurg.2022.1071363

**Published:** 2023-01-09

**Authors:** Jiu-Heng Yin, Yi-Hui Chen, Yan-Bei Ren, Rong Wang, Shuai Su, En-Lai Jiang, Yun-Bo Li, Ting Wang, Wei-Dong Xiao, Guang-Sheng Du

**Affiliations:** ^1^ Department of General Surgery, Xinqiao Hospital, Army Medical University, Chongqing, China; ^2^ Nursing Department, Nursing School of Chongqing Medical and Pharmaceutical College, Chongqing, China

**Keywords:** gastric cancer, single-incision plus one-port laparoscopic total gastrectomy (SITG + 1), Overlap esophagojejunostomy, total gastrectomy, minimally invasive technique

## Abstract

**Background:**

This study aimed to explore the feasibility and safety of single-incision plus one-port laparoscopic total gastrectomy (SITG + 1) with Overlap esophagojejunostomy (SITG + 1-Overlap) and to share preliminary experiences.

**Methods:**

This retrospective study included 10 patients with gastric cancer located in the cardia or body who underwent SITG + 1-Overlap between August 2020 and October 2021.The demographics, tumor characteristics, postoperative outcomes, and short-term complications of all the enrolled patients were summarized and statistically analyzed. Data were expressed as mean ± standard deviation (SD) if they were normally distributed. Otherwise, Median (Quartile1, Quartile3) was used.

**Results:**

In the collective perioperative data of these 10 patients who underwent radical gastrectomy, the median of the length of transumbilical incision and blood loss were 3.0 cm and 100.0 ml respectively, and the mean operation time and 385.5 ± 51.6 min. Postoperative data indicated that the gastric tube was removed on 2.0 (2.0, 3.0) days, and the timing of first feeding, activity, flatus, and defecation was 1.5 (1.0, 2.0) days, 2.0 (2.0, 2.0) days, 3.0 (2.0, 3.0) days, and 3.8 ± 0.6 days, respectively. The timing of drainage tube removal was 4.6 ± 1.0 days after operation. The duration of hospital stay was 7.5 ± 1.2 days and the VAS pain scores for the 3 days following surgery were 3.0 (2.0, 3.3), 2.0 (2.0, 3.0), and 1.5 (1.0, 2.0) respectively. The mean number of retrieved lymph nodes was 30.7 ± 13.2. Most biochemical indicators gradually normalized with the recovery of the patients after surgery. No 30-day postoperative complications were noted.

**Conclusions:**

For the first time, our preliminary data indicate the feasibility and safety of Overlap esophagojejunostomy in SITG + 1 surgery. This modified Overlap procedure has the potential to simplify the reconstruction procedure and lower the technical challenge of SITG + 1 radical gastrectomy for cardia or upper gastric cancer in the early and advanced stages.

## 1. Introduction

As a novel, minimally invasive technique, laparoscopic surgery has become the primary treatment for gastric cancer (1). Furthermore, new emerging techniques have been developed to reduce the invasiveness of laparoscopic surgery (2). In recent years, single-incision laparoscopic surgery (SILS) has emerged as a popular research topic (3). The SILS technique takes full advantage of the innate fold of the umbilicus. The vertical endoscope operation channel significantly improved the postoperative cosmetic appearance of the abdominal wall and reduced the surgical trauma. The SILS technique has been used in gastric cancer surgery, and the number of case reports in this field is increasing. However, most studies on SILS have focused on distal gastric cancer, and the application of total gastrectomy has only been sporadically reported, mainly because of the difficulty of performing radical total gastrectomy and subsequent esophagojejunostomy under a single incision (4).

As an alternative method to increase the feasibility and reduce the technical challenges of pure single-incision laparoscopic gastrectomy, the single-incision plus one-port laparoscopic gastrectomy (SILG + 1) technique has been gradually adopted by an increasing number of surgical teams in recent years (5, 6). We have already demonstrated the great potential of SILG + 1 procedures in radical surgery for gastric cancer in both early and advanced stages (7). The shorter incision length, improved postoperative pain, and similar postoperative complication rates fully demonstrate the advantages of the SILG + 1 procedure over the conventional 5-port laparoscopic procedure. The better cosmetic score and similar cosmetic effect after month postoperatively display the unique advantage of a single incision procedure. Moreover, for the first time, a π-shaped anastomosis, named SILT-π, was introduced to overcome the technical challenges and simplify the esophagojejunal reconstruction procedure after single-incision plus one-port laparoscopic total gastrectomy (SITG + 1).

It is noteworthy that the unique characteristics of “pre-pulling and latter transection” in π-shaped anastomosis have its own limitations as compared with other reconstruction methods: once the upper esophageal resection margin of the intraoperative frozen section is positive after π-shaped esophagojejunostomy, it will be more challenging for the surgeon to re-perform the esophagojejunostomy in the higher position after the extended resection of the adjacent esophagus, especially in SITG + 1 conditions. Therefore, new reconstruction methods are needed for esophagojejunostomy, especially for cardia cancer with a relatively higher location and poorly defined upper margin on endoscopic examination. The Overlap method for esophagojejunostomy was introduced by Inaba et al. in 2010 (8). This Overlap anastomosis renders the positions of the esophagus and jejunum consistent with the direction of the intestinal peristalsis, which was already well documented, with the lowed incidence of anastomotic-related complications, such as mesenteric tension, anastomotic stricture and leakage (9–11). Moreover, the “pre-transection and latter anastomosis” design of the Overlap method avoids the obvious limitation of the π-shaped anastomosis, considering the possibility of a positive upper resection margin. We retrospectively analyzed the short-term outcomes of 10 patients who underwent SITG + 1 with Overlap esophagojejunostomy (SITG + 1-Overlap), evaluated its feasibility and safety, and summarized the preliminary experience.

## 2. Materials and methods

### 2.1. Patients

Ten male patients with gastric cancer who underwent SITG + 1-Overlap surgery between August 2020 and October 2021 at the Xinqiao Hospital of the Army Medical University were included in our study. The criteria for eligibility included age within 18–80 years old, a preoperative pathological diagnosis of gastric cancer, a clinical tumor stage of T1-4N1-3M0, BMI within 18–27 kg/m^2^. Exclusion criteria included pathological stage IV gastric cancer, neoadjuvant chemotherapy history, and history of severe heart, liver, lung, or kidney dysfunction. All procedures were performed in accordance with the ethical standards of the Committee on Human Experimentation (China Registered Clinical Trial Ethics Review Committee No. chiECRCT-201701109). Informed consent was obtained from all patients. The tumor-node-metastasis (TNM) stage was determined based on the eighth edition of the American Joint Committee on Cancer (AJCC) staging manual.

### 2.2. Surgical technique

#### 2.2.1. SITG + 1 with D1+ or D2 lymph node dissection

**Figure 1:**
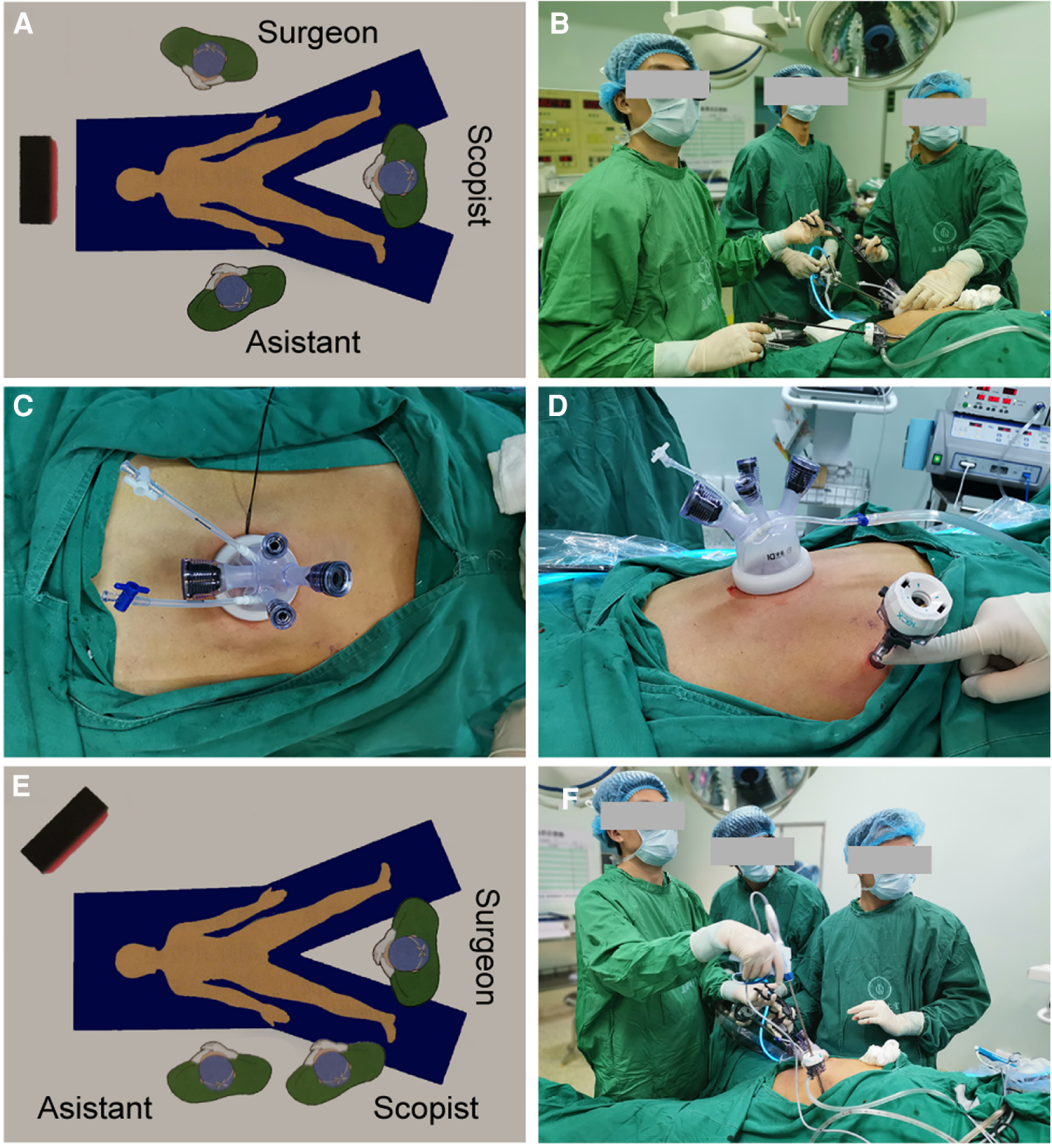
(**A,B**) Diagram illustrating the surgical field setup at the beginning of the surgery. (**C,D**) A commercial four-hole wound-protecting device was inserted into a transumbilical incision, and an 12-mm additional assistant trocar was placed as an auxiliary operating hole 2 cm below the costal margin of the anterior axillary line in the upper left abdomen. (**E,F**) When the surgeon cleaned the lymph nodes on the left side of the greater curvature of the stomach, the surgeon moved from the patient's left side to between the patient's legs, with the first assistant and the other assistant holding the lens while standing on the right side of the patient.

**Figure 2:**
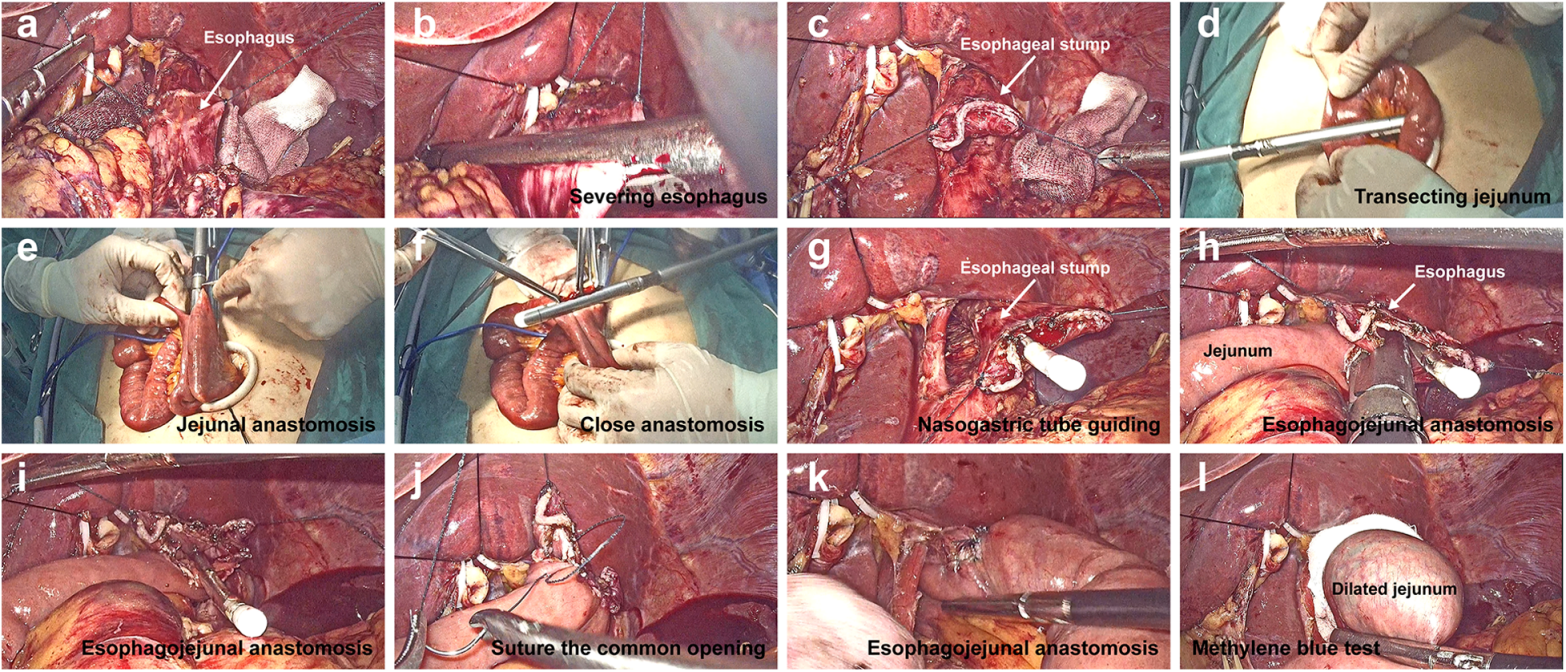
Digestive tract reconstruction in single-incision plus one-port laparoscopic gastrectomy (SILG + 1). (**A–C**) The left lobe of the liver was overhung using a percutaneous 2-0 nylon purse-string suture and hemolok ligation clip. Suturing was performed *via* a stitch with a 4-0 barbed line on the left and right sides of the pre-separation esophagus. The esophagus and stomach were transected using a linear stapler. (**D–F**) a side-to-side jejunal anastomosis was created using the stapler between the afferent jejunum and a point 40 cm below the efferent jejunum. (**G**) After opening a hole in the middle of the esophageal stump, the gastric tube was pulled out from the hole to guide the correct lumen. (**H,I**) a side-to-side esophagojejunal anastomosis (Overlap) was performed. (**J,K**) A 4-0 barbed line was used to close the common opening, and another 4-0 barbed line was used to reinforce the anastomotic stoma by suturing the seromuscular layer. (**L**) Methylthionine chloride was injected through the gastric tube to detect the integrity of the anastomosis.

Here, we describe the SITG + 1-Overlap with the D1+ or D2 lymph node dissection procedure. Briefly, the patient was placed in a supine reverse Trendelenburg position. The surgeon and assistant stood on the left and right sides of the patient, respectively, while the scopist stood between the patient's legs (Figures 1A,B). A commercial four-hole wound-protecting device was then inserted into a transumbilical incision measuring 2.5–5.0 cm (Figure 1C). The abdominal cavity was insufflated with carbon dioxide and a 10-mm three-dimensional high-definition scope was inserted *via* a 12-mm hole in the wound-protecting device. Separately, an 12-mm additional assistant trocar was placed as an auxiliary operating hole, 2 cm below the costal margin of the anterior axillary line in the upper left abdomen (Figure 1D). After the left lobe of the liver was overhung using a percutaneous 2-0 nylon purse-string suture (one end of suture was secured to the abdomen; the another was secured to the dissected gastrohepatic ligament with a 2–3 hemolok ligation clip) (Figure 2A), we performed routine total gastrectomy with D1 + or D2 lymph node dissection, including partial omentectomy. When the surgeon cleaned the lymph nodes on the left side of the greater curvature of the stomach, the surgeon moved from the patient's left side to between the patient's legs, with the first assistant and the other assistant holding the lens while standing on the patient's right side (Figures 1E,F).

#### 2.2.2. Intracorporeal Overlap esophagojejunostomy

The specific steps of this procedure are illustrated in Figure 2. Briefly, the lower esophagus was fully dissociated along its periphery. The Overlap anastomosis technique was used to create side-to-side esophageal and jejunal anastomoses. In this technique, the pre-separation plane of the lower esophagus is first determined according to the upper margin of the tumor. Suturing was performed *via* a stitch with a 4-0 barbed line on the left and right sides of the pre-separation esophagus. The assistant pulled the barbed suture and the surgeon pulled the stomach downward with the left hand. The esophagus and stomach were transected using a linear stapler through an additional auxiliary hole (Figures 2B,C). The surgeon closed the pneumoperitoneum, removed the umbilical wound-protecting device, and removed the entire stomach specimen.

After a jejunal loop located approximately 30 cm distal to the Treitz ligament was taken out and transected using a linear stapler outside the abdominal cavity (Figure 2D), a side-to-side jejunal anastomosis was created using a stapler between the afferent jejunum and a point 40 cm below the efferent jejunum (esophagojejunal anastomosis) and common opening was closed using a stapler (Figures 2E,F). After the mesenteric hiatus was closed, the bowel was inserted into the abdominal cavity, and pneumoperitoneum was re-established. To facilitate esophagojejunal anastomosis, the diaphragmatic angles on both sides were cut appropriately to provide space for the anastomosis. After opening a hole in the middle of the esophageal stump, the gastric tube was pulled out to guide the correct lumen (Figure 2G). One fork of linear stapler was inserted through a hole 7 cm from the efferent jejunum stump, and another fork was inserted into the hole in the esophageal stump along the gastric tube, in the process of which the 4-0 barbed line was used to help pull the esophagus. A side-to-side esophagojejunal anastomosis was created (Figures 2H,I). A 4-0 barbed suture reserved in the stump of the esophagus was used to close the common opening, and another 4-0 barbed suture reinforced the anastomosis by suturing the seromuscular layer (Figures 2J,K). The gastric tube was placed at the anasomotic site and the distal jejunum was clipped using laparoscopic forceps. Methylthionine chloride was injected into the gastric tube to determine the integrity of the anastomosis (Figure 2L).

### 2.3. Data collection and statistical analysis

We recorded basic data on age, sex, body mass index (BMI), ASA score, clinical stage, and tumor location. Surgical data included incision length, operative time, intraoperative blood loss, and intraoperative blood transfusion. Postoperative data were also recorded, including VAS pain score, timing of gastric tube removal, first feeding, activity, flatus, defecation, duration of hospital stay, and any complications. Postoperative pathology included tumor size and differentiation, proximal and distal resection margin distances, number of dissected lymph nodes, and TNM stage. Perioperative biochemical indices were recorded separately. Data were analyzed using SPSS software (version 20.0; SPSS, Chicago, IL, United States). Data were expressed as mean ± standard deviation (SD) if they were normally distributed. Otherwise, median (Quartile1, Quartile3) was used.

## 3. Results

### 3.1. Patients' information and clinical characteristics of tumors

The basic information of the enrolled patients and the clinical characteristics of the tumors are summarized in Table 1. All patients were male, and their ages and BMI were 61.8 ± 8.2 years and 19.9 (18.0, 27.0) kg/m^2^ respectively. Tumor locations included six in the gastric body and four in the cardia of the stomach. The preoperative clinical stage ranged from cT1N0M0 to cT4N3M0.

**Table 1:** Patients’ basic information and clinical characteristics of tumor.

Characteristics	Case 1	Case 2	Case 3	Case 4	Case 5	Case 6	Case 7	Case 8	Case 9	Case 10	Mean ± SD/Median (Q1, Q3)
Gender (Male/Female)	Male	Male	Male	Male	Male	Male	Male	Male	Male	Male	–
Age (years)	65	50	61	48	62	67	58	65	66	76	61.8 ± 8.2
BMI (kg/m^2^)	26.4	18.7	18.0	25.7	20.7	19.0	18.9	27.0	23.4	18.6	19.9 (18.7, 25.9)
ASA Score	II	II	II	II	III	II	II	II	II	III	–
Clinical stage (M0)	cT4N1	cT4Nx	cT1N0	cT4N0	cT4N3	cT1N0	cT4N0	cT1N0	cT3N3	cT3N3	–
Tumor location	cardia	body	body	cardia	body	body	body	body	cardia	cardia	–

BMI, body mass index; ASA Score, american society of anesthesiologists score; Clinical stage is according to AJCC 8th edition; Q1, Quartile1; Q3, Quartile3.

### 3.2. Perioperative situations and postoperative pathological examination

**Figure 3:**
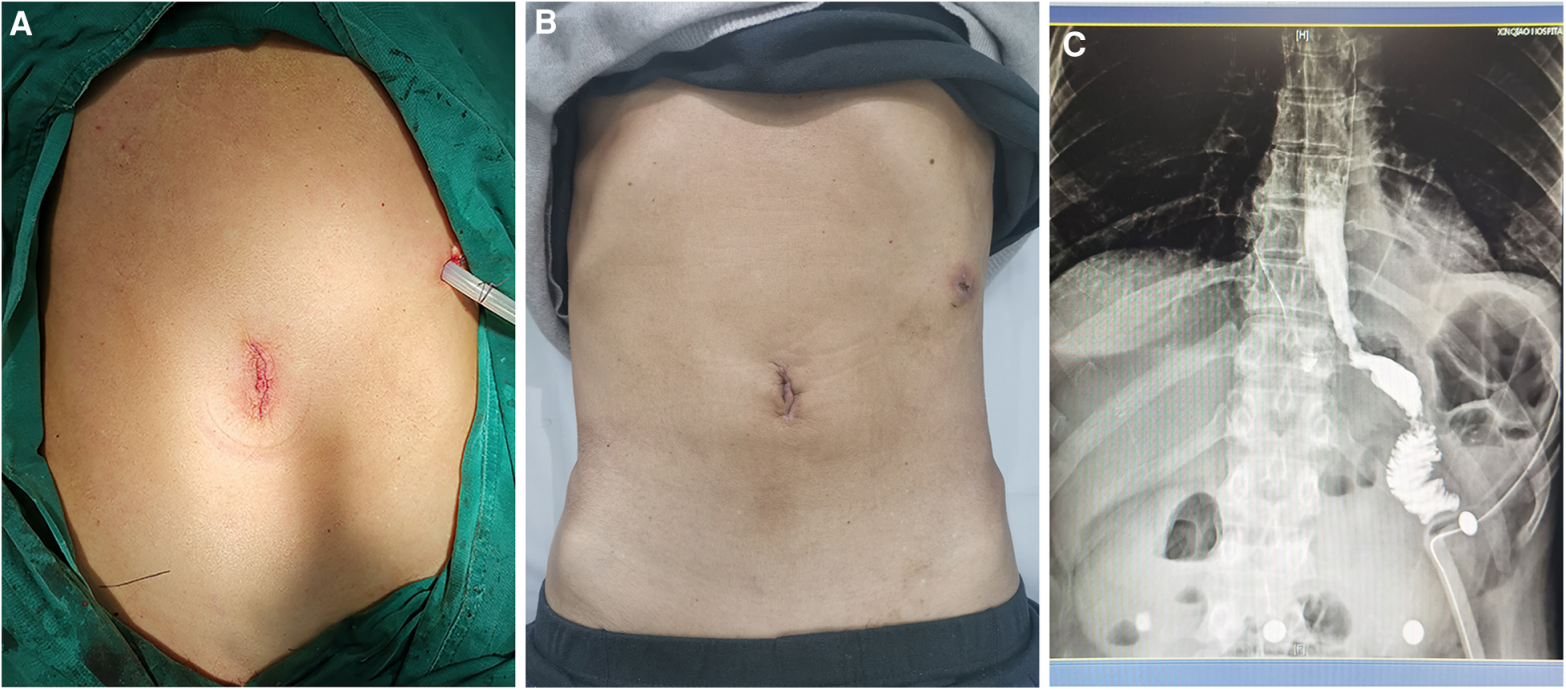
(**A,B**) Small incision around the umbilicus is shown on the day of surgery vs. day 21 after surgery. (**C**) Patency of the anastomosis was detected using barium meal examination.

The intraoperative and postoperative data are presented in Table 2. The length of the surgical incision was 3.0 (2.5, 3.3) cm, and the total operation time was 385.5 ± 51.6 min. The intraoperative blood loss was 100.0 (50.0, 162.5) ml during their operations. A small incision around the umbilicus seems to be more aesthetic (on the day of surgery vs. day 21 after surgery) (Figures 3A,B). There were no any intraoperative adverse events.

**Table 2:** Perioperative situations and postoperative pathological examination.

Characteristics	Case 1	Case 2	Case 3	Case 4	Case 5	Case 6	Case 7	Case 8	Case 9	Case 10	Mean ± SD/Median (Q1, Q3)
Operation duration (min)	315.0	295.0	425.0	460.0	425.0	385.0	415.0	405.0	360.0	370.0	385.5 ± 51.6
Incision length (cm)	3.0	2.5	2.5	3.0	3.0	4.0	3.0	5.0	2.5	3.0	3.0 (2.5, 3.3)
Blood lose (ml)	100.0	100.0	50.0	150.0	50.0	200.0	150.0	100.0	50.0	200.0	100.0 (50.0, 162.5)
Intraoperative complications	no	no	no	no	no	no	no	no	no	no	–
Nasogastric Tube Removal (days)	2.0	2.0	2.0	3.0	3.0	3.0	3.0	2.0	2.0	2.0	2.0 (2.0, 3.0)
First feeding (days)	1.0	2.0	2.0	1.0	2.0	2.0	3.0	1.0	1.0	1.0	1.5 (1.0, 2.0)
First activity (days)	2.0	3.0	2.0	2.0	2.0	2.0	2.0	2.0	2.0	2.0	2.0 (2.0, 2.0)
First Flatus (days)	3.0	3.0	2.0	2.0	3.0	3.0	3.0	3.0	2.0	2.0	3.0 (2.0, 3.0)
First Defecation (days)	4.0	5.0	4.0	5.0	5.0	6.0	5.0	4.0	3.0	4.0	3.8 ± 0.6
Drainage Tube Removal (days)	4.0	6.0	4.0	5.0	5.0	6.0	5.0	4.0	3.0	4.0	4.6 ± 1.0
Hospital Stay (days)	6.0	7.0	8.0	7.0	8.0	8.0	10.0	6.0	8.0	7.0	7.5 ± 1.2
VAS score											
POD 1	3.0	4.0	2.0	4.0	3.0	2.0	2.0	3.0	2.0	3.0	3.0 (2.0, 3.3)
POD 2	2.0	3.0	2.0	2.0	3.0	2.0	2.0	3.0	3.0	2.0	2.0 (2.0, 3.0)
POD 3	2.0	2.0	1.0	2.0	2.0	1.0	1.0	1.0	2.0	1.0	1.5 (1.0, 2.0)
Complications	no	no	no	no	no	no	no	no	no	no	–
Tumor cell differentiation	P	P	P	P	P	M	M	M	M	H	–
Proximal edge (cm)	0.5	5.0	3.0	2.0	4.5	3.0	5.0	6.0	1.0	1.0	3.1 ± 2.0
Distal edge (cm)	10.0	7.0	10.0	12.0	7.0	9.5	9.5	1.2	8.0	10.0	8.4 ± 3.0
Positive LNs	0	7	0	0	12	13	0	0	0	8	–
Retrieved LNs	17	24	18	14	30	40	54	47	41	22	30.7 ± 4.4
Tumor size (maximum diameter, cm)	4.2	3.5	3.0	1.5	4.0	3.5	1.0	4.3	3.0	3.5	3.2 ± 0.3
Pathological stage (M0)	pT4N0	pT4N3	pT1N0	pT4N0	pT4N3	pT4N3	pT1N0	pT4N0	pT1N0	pT4N3	–

POD, days postoperation; P, poorly differentiated; M, moderately differentiated; H, high differentiated; Q1, Quartile1, Q3, Quartile3.

Regarding the recovery process, the timing of the first feeding, activity, flatus, defecation, and duration of postoperative hospital stay are recorded in Table 2. The gastric tube was removed 2–3 days after surgery, and the abdominal drainage tube was removed 3–6 days after surgery. The timing of first exhaust was 3.0 (2.0, 3.0) days, and the timing of first defecation were 3.8 ± 0.6 days. The VAS pain scores were 3.0 (2.0, 3.3), 2.0 (2.0, 3.0), and 1.5 (1.0, 2.0) on POD1, 2 and 3 respectively. The postoperative hospital stay was 7.5 ± 1.2 days. Noteworthily, Patient 7 already met the discharge criteria on day 6 after surgery, but the outbreak of COVID-19 infection led to a prolonged hospital stay. The patency of the anastomosis was determined by barium meal examination (Figure 3C). No 30-day postoperative complications were noted.

Postoperative pathological results analysis recorded in Table 2 and showed that the proximal surgical margin was 3.1 ± 2.0 cm and the distal margin was 8.4 ± 3.0 cm. The number of dissected lymph nodes was 30.7 ± 13.2. Postoperative pathological stages ranged from pT1N0M0 to pT4N3M0.

### 3.3. Perioperative biochemical indicators

Perioperative biochemical indicators, including White Blood Cells (WBC), hemoglobin (Hb), procalcitonin (PCT), aspartate transaminase (AST), Creatinine, Prealbumin and Albumin, are shown in Table 3. These indicators were collected preoperatively and on POD 1, 3, and 5 days after surgery. Most biochemical indicators gradually normalized with the recovery of the patients after surgery. However, the prealbumin level was relatively low on POD 1, 3, and 5. Two patients had significantly abnormal liver function on postoperative first day, which may be related to intraoperative liver overhung.

**Table 3:** Perioperative biochemical indicators.

Characteristics	Case 1	Case 2	Case 3	Case 4	Case 5	Case 6	Case 7	Case 8	Case 9	Case 10	Mean ± SD/Median (Q1, Q3)
WBC (10^9^)											
pre-operation	5.9	4.2	4.9	4.1	4.4	3.6	4.5	4.3	6.3	4.0	4.6 ± 0.3
POD1	13.0	11.4	9.6	12.4	8.2	7.1	9.8	13.2	11.6	6.2	10.3 ± 0.8
POD3	6.4	7.9	5.9	5.2	6.4	7.0	9.9	15.0	7.7	7.6	7.3 (6.3, 8.4)
POD5	7.4	5.2	4.9	12.7	4.2	6.1	7.3	9.8	6.2	6.0	7.0 ± 0.8
Hb (g/L)											
pre-operation	140.0	110.0	132.0	120.0	90.0	92.0	168.0	137.0	143.0	100.0	123.2 ± 8.0
POD1	144.0	101.0	144.0	126.0	82.0	97.0	130.0	113.0	142.0	111.0	119.0 ± 6.8
POD3	128.0	88.0	117.0	122.0	88.0	94.0	117.0	110.0	121.0	92.0	107.7 ± 4.9
POD5	139.0	94.0	119.0	119.0	96.0	94.0	118.0	118.0	116.0	95.0	110.4 ± 5.0
PCT (ng/L)											
POD1	0.1	1.2	0.2	0.4	0.4	0.7	0.4	0.8	1.8	1.0	0.7 ± 0.2
POD3	0.1	1.0	0.7	0.9	0.2	0.5	1.6	0.4	1.7	0.6	0.8 ± 0.2
POD5	0.1	0.2	0.3	0.4	0.1	0.1	0.6	0.1	0.7	0.4	0.3 ± 0.1
AST (U/L)											
pre-operation	18.0	28.3	16.7	29.7	17.1	20.4	43.9	19.5	24.1	11.4	22.9 ± 2.9
POD1	161.8	207.9	281.7	93.8	85.6	146.7	268.5	157.3	108.6	448.4	196.0 ± 35.2
POD3	20.3	42.0	95.3	18.2	82.6	25.3	77.7	39.7	37.8	63.9	50.3 ± 8.8
POD5	28.6	30.2	33.0	15.9	35.7	15.7	36.6	80.2	33.9	23.9	31.6 (21.9, 35.9)
Creatinine (umol/L)											
pre-operation	64.4	60.7	56.9	93.7	69.8	68.7	68.2	66.4	77.2	50.8	67.7 ± 3.7
POD1	60.0	72.2	48.3	77.0	64.7	62.5	80.6	68.4	81.3	43.1	65.8 ± 4.1
POD3	63.7	65.8	47.0	111.8	58.9	62.1	65.7	51.9	80.5	42.4	62.9 (50.7, 69.5)
POD5	68.7	76.9	41.7	86.5	72.6	63.7	57.6	53.2	73.9	37.9	63.3 ± 4.9
Prealbumin (mg/L)											
pre-operation	256.0	145.0	234.0	200.0	195.0	174.0	432.0	223.0	255.0	115.0	222.9 ± 27.4
POD1	208.0	168.0	167.0	172.0	127.0	172.0	234.0	198.0	186.0	107.0	173.9 ± 11.7
POD3	114.0	118.0	71.0	75.0	106.0	95.0	158.0	124.0	83.0	34.0	97.8 ± 10.8
POD5	183.0	161.0	94.0	51.0	131.0	112.0	145.0	146.0	101.0	56.0	121.5 (94.0, 161.0)
Albumin (mg/L)											
pre-operation	42.3	29.9	44.7	33.8	41.1	41.2	44.5	40.4	45.9	35.8	40.0 ± 5.2
POD1	37.8	31	37.2	32.9	31.3	33.9	30.1	31.6	38.9	29.4	33.4 ± 3.4
POD3	30.5	31.6	31.2	29.7	34.3	31.1	31.8	32.4	35.0	30.2	31.8 ± 1.7
POD5	42.8	40.7	31.9	31.3	37.2	30.7	33.8	32.8	34.7	37.2	35.3 ± 4.1

POD, days postoperation; WBC, white blood cell; Hb, hemoglobin; PCT, procalcitonin; AST, aspartate transaminase; Q1, Quartile1, Q3, Quartile3.

## 4. Discussion

Reduced-port laparoscopic surgery (RPS) and single-incision laparoscopic surgery (SILS) have become increasingly popular (12). As an alternative method to increase the feasibility and reduce the technical challenges of pure SILS, the single-incision plus one-port laparoscopic surgery (SILS + 1) technique has been gradually applied by an increasing number of surgical teams in recent years (13, 14). Regarding the application of SITG + 1, most studies have only observed the short-term efficacy of SITG + 1 in distal early gastric cancer (5, 15, 16). Based on our clinical experience and on improvements in our technique, single-incision plus one-port laparoscopic total gastrectomy (SITG + 1) has been proven to be feasible and safe for radical resection of early and advanced gastric cancer (7). However, SITG + 1 is difficult to create a good surgical field because surgical instruments interfere with each other through a single incision. Owing to the narrow field of view, the doctor's operating space can be affected, leading to difficulties in constructing the digestive tract (17, 18). Additionally, the surgical procedure is complex and requires experienced surgeons. Unexpected adverse events can be difficult to manage intraoperatively.

In 2022, our study extended the indication of the SITG + 1 technique to advanced gastric cancer, particularly total gastrectomy (7). SITG + 1 combined with esophagojejunal π-shaped anastomosis (SITG-π) has been introduced to overcome technical challenges and simplify esophagojejunal anastomosis after total gastrectomy. Moreover, a good long-term outcome will be published recently, according to a 3-year follow-up study (unpublished data). However, we noticed some drawbacks of the SITG-π anastomosis. A fatal disadvantage of this method is that the tumor margin can only be checked after anastomosis, leading to a hidden danger of a positive margin. Once intraoperative freezing results in a positive esophageal margin, the surgeon needs to enlarge the resection of the esophagus under SITG + 1 and re-perform esophagojejunal anastomosis at a higher position, which can be challenging. Additionally, an esophagojejunal π-shaped anastomosis may lead to an antiperistalsis at the junction of the esophagus and jejunum, which is not conducive to esophageal emptying (19).

It is worth noting that a new esophagojejunal anastomosis (Overlap) can avoid the drawbacks of SITG-π. However, it is not clear whether SITG + 1 combined with esophagojejunal Overlap anastomosis (SITG + 1-Overlap) is feasible and safe for surgical treatment of early and advanced gastric cancer. In this study, 10 patients with early or advanced gastric cancer underwent SITG + 1-Overlap surgery. All procedures were performed successfully without any intra- or postoperative anastomosis-related complications. All esophageal resection margins were negative, and conversion to open surgery was not required. None of the patients showed any obvious postoperative choking. The feasibility and safety of SITG + 1-Overlap in the treatment of early and advanced gastric cancers were preliminarily confirmed. To the best of our knowledge, the present study is the first to report the introduction of the Overlap esophagojejunostomy in SITG + 1 procedures.

Esophagojejunal anastomosis is a key step in SITG + 1 for gastric cancer, which is difficult to perform using staplers or sutures under the limited laparoscopic view available. The Overlap anastomosis of the esophagus and jejunum is in the isoperistaltic direction, which is more in line with the normal physiological structure and is conducive to esophageal emptying. Wang et al. believed that reverse peristaltic anastomosis might lead to a physiological barrier in gastrointestinal continuity (20). In addition, the common opening was closed securely with hand sutures after creating an access opening hole using a linear stapler. This technique rarely results in anastomotic narrowing because of large triangular anastomosis and hand sutures (21). Finally, π-shaped anastomosis is difficult for gastric cardia cancer at a high position, especially in patients with fat bodies and a short mesentery. A higher esophagojejunal Overlap anastomosis can be performed due to the distal tension-free jejunum.

However, esophagojejunal Overlap anastomosis has some shortcomings. The complex closure with hand sutures during SITG + 1 requires a higher degree of surgical skill and takes longer time to perform, which is not suitable for beginners. To overcome these issues, we modified the procedure. First, for easier suturing of the common hole, the addition of an auxiliary port can effectively prevent instrument collisions and reduce the difficulty in stapling and suturing. Secondly, before the esophagus was cut off, the pre-separation plane was marked in advance, above which two knotless unidirectional barbed sutures were stitched on the left and right sides of the esophagus. Sutures enabled the surgeon to pull the separated esophagus to avoid effectively esophageal slippage, even within the deep area. The assistant lifted the two barb sutures upward, and the surgeon pulled the esophagus downward with his left hand and entered a linear stapler from the auxiliary hole to cut the esophagus with his right hand. After anastomosis was created, barbed sutures were directly used to suture the common opening. Third, a nasogastric tube was pulled out of the stump as a guide to identify the true lumen of the esophagus. A stay suture was then placed to avoid a false gap between the esophageal mucosa and wall.

## 5. Conclusions

The feasibility and safety of the SITG + 1-Overlap in early and advanced gastric cancers were confirmed in our study. SITG + 1-Overlap can be performed by experienced surgeons because of isperistalsis and less anastomotic stenosis despite its long operative time. Despite the very small number of cases without a control group, the present study shares the preliminary technical experience of SITG + 1-Overlap. The long-term outcomes were not evaluated in the present study. Therefore, large-scale RCT should be conducted to obtain a higher grade of evidence. Taken together, this study provides new options for surgeons who perform total gastrectomy under total laparoscopy.

## Data Availability

The original contributions presented in the study are included in the article/Supplementary Material, further inquiries can be directed to the corresponding author/s.
